# The Link between the Nature of the Human–Companion Animal Relationship and Well-Being Outcomes in Companion Animal Owners

**DOI:** 10.3390/ani14030441

**Published:** 2024-01-29

**Authors:** Annalyse Ellis, Sarah C. E. Stanton, Roxanne D. Hawkins, Steve Loughnan

**Affiliations:** 1 Department of Psychology, School of Philosophy, Psychology and Language Sciences, University of Edinburgh, Edinburgh EH8 9AD, UK; sarah.stanton@ed.ac.uk (S.C.E.S.); steve.loughnan@ed.ac.uk (S.L.); 2 Department of Clinical and Health Psychology, School of Health in Social Science, University of Edinburgh, Edinburgh EH8 9AG, UK; roxanne.hawkins@ed.ac.uk

**Keywords:** pets, companion animals, well-being, attachment, mental health, self-expansion, responsiveness, insensitivity

## Abstract

**Simple Summary:**

Past research regarding the impact of companion animals on well-being has yielded variable results, with some studies finding that companion animals have a positive impact on mental well-being and others finding neutral or negative impacts. This study explored potential causes for these contradictory results, measuring the relationship science concepts of attachment, self-expansion, perceived responsiveness, and perceived insensitivity within the human–companion animal relationship, as well as mental health outcomes of depression, anxiety, and affect; and loneliness as a mediator between the relationship science concepts and the mental health outcomes. Attachment, self-expansion, and perceived pet insensitivity all significantly predicted at least one mental health outcome. We also found that loneliness mediates the relationship between attachment, self-expansion, and perceived pet insensitivity, and all of the mental health outcomes. Our results indicate that these relationship dimensions play a role in the well-being benefits derived from having a companion animal.

**Abstract:**

Research into the impact of companion animals on well-being has been both extensive and inconclusive, with studies finding both positive and negative relationships. The present research explored three previously unexamined relationship science concepts that may help clarify whether companion animals provide well-being benefits: self-expansion (the process of adding positive content to the self through incorporating new resources and perspectives into one’s identity or engaging in novel, exciting activities), perceived pet responsiveness, and perceived pet insensitivity; as well as attachment. We focused on dog and cat owners’ depression, anxiety, positive and negative affect, and loneliness through an online survey with a large sample population (*N* = 1359). We found that perceived pet insensitivity is a significant positive predictor of depression, anxiety, negative affect, and loneliness; that attachment is a significant positive predictor of depression, anxiety, and loneliness, and a significant negative predictor of positive affect; and that self-expansion is a significant positive predictor of positive affect, and a significant negative predictor of loneliness. Loneliness emerged as a mediator in the relationship between perceived pet insensitivity, attachment, self-expansion, and all mental well-being outcome variables. These findings indicate that perceived pet insensitivity, attachment, and self-expansion may play an important yet neglected role in well-being outcomes.

## 1. Introduction

Poor mental well-being is a significant and common concern; around one in eight adults live with a mental health problem [1]. Referrals to mental health services are increasing [2], and around 703,000 individuals die by suicide each year globally [3]. Social relationships are credited with improving well-being, decreasing loneliness, and decreasing mortality risk [4,5,6]. Central amongst these relationships may be peoples’ close relationships with their companion animals. More than 50% of homes worldwide have companion animals [7], and many of these companion animal owners report that their companion animal contributes significant social support [8], providing a source of help when their mental health is poor or during emotional distress [9,10,11]. Although considerable research has examined the impact of companion animals on mental health and well-being, the results have been variable and inconclusive [12,13,14]. 

The present research aims to understand the relationship between companion animal ownership and companion animal owners’ mental well-being by exploring the nature of the human–animal relationship. Specifically, we examine three well-established relationship science concepts: self-expansion [15], perceived pet responsiveness and insensitivity [16], and attachment [17]. Self-expansion refers to the incorporation of new positive content, resources, and perspectives into one’s identity, leading to growth in one’s self-efficacy [15]. Perceived pet responsiveness and insensitivity denotes the extent to which a companion animal owner believes their companion animal to be responsive and receptive, or conversely insensitive and unreceptive to their needs [16]. Lastly, attachment refers to the bond between the companion animal owner and companion animal [17]. While self-expansion and perceived responsiveness and insensitivity have been robustly shown to predict well-being in romantic and close human relationships, these concepts have not yet been applied to understanding people’s relationships with their companion animals. We hypothesized that perceived pet responsiveness, self-expansion, and attachment would be associated with lower depression, anxiety, and negative affect levels, and higher positive affect levels; while perceived pet insensitivity would be associated with the inverse results. We also hypothesized that loneliness would mediate the relationships between all relationship science concepts and all mental well-being outcomes.

### 1.1. Past Research on Companion Animal Ownership and Mental Well-Being

There have been several studies that have found that companion animal ownership benefits well-being. Companion animal owners report lower levels of anxiety [18] and depression [19], and more positive affect [20]. These effects are especially pronounced amongst vulnerable groups including the elderly [21,22], people living alone [23], and people living with life-threatening and life-limiting diseases (e.g., HIV-AIDS) [24]. Companion animals can also be a powerful source of mental well-being during periods of crisis, with companion animal owners often reporting that they find comfort in their companion animals during times of distress [11]. For example, adults with companion animals experienced smaller increases in loneliness and smaller decreases in mental well-being during the COVID-19 pandemic [25], and also reported more positive emotions, and better psychological well-being and coping self-efficacy [26]. In short, there is evidence that companion animals can benefit well-being. 

However, a smaller but still substantial body of evidence indicates that companion animal ownership may have no impact or even have a negative impact on well-being. During the COVID-19 pandemic, companion animal owners were equally anxious compared with non-companion animal owners [27]. Companion animal ownership has been found to have no impact on depression or anxiety in carefully matched-samples designs [28]. Likewise, longitudinal studies have found no evidence that companion animals reduce loneliness [29], nor that they increase general well-being [30]. Indeed, companion animal ownership has been linked to *higher* levels of mental illness, especially depression and anxiety [31,32]. These effects are also observed amongst vulnerable populations such as the elderly [33]. In essence, there is no consistent picture of the relationship between companion animals and well-being. 

The link between companion animal ownership and well-being requires further exploration, and there are several potential explanations for the variability in results. Past research has indicated that sociodemographic differences, such as socioeconomic status, health, living arrangements, and employment status, between companion animal owners and non-companion animal owners can account for some of this variability [34,35]. Additionally, some of the variability may be caused by small sample sizes, homogeneous samples, lack of consistency in research design, and small effect sizes [36]. Finally, few studies have examined the mechanisms that underpin the relationship between human–animal relationships and well-being, apart from attachment bond, which has been found to be related to mental health outcomes. We suggest that other human–animal relationship processes, such as self-expansion, perceived pet responsiveness, and perceived pet insensitivity, represent an important yet neglected piece of this puzzle.

### 1.2. Self-Expansion

Self-expansion theory argues that individuals are motivated to expand their sense of self through incorporating new resources and perspectives into their identities [15]. There are two main elements to the self-expansion model: (1) that people are motivated to self-expand to increase their sense of efficacy, which makes them better able to reach their goals, and (2) that self-expansion can take place through forming close relationships and including others’ resources, identities, and perspectives into the self [37]. 

There are several ways in which self-expansion may contribute to improved mental well-being outcomes. The process of self-expansion itself can feel enjoyable [37]. Beyond this initial rush, individuals are motivated to self-expand in order to increase their sense of efficacy and their ability to achieve their goals, and this sense of efficacy and success is linked to more positive well-being [37,38]. This positive relationship extends beyond simply feeling better to encompassing reductions in poor mental health. Self-expansion can reduce depressive symptoms directly [39]. It can also help improve and clarify the self-concept [40,41], which is associated with lower levels of anxiety and depression [42]. Further, self-expansion leads to improved relationship quality [43], which is also associated with lower levels of depression and anxiety [44]. In short, self-expansion can both directly and indirectly lead to improved mental well-being.

Although the research discussed above involves human–human relationships, self-expansion can also occur through other modalities, such as engaging in activities and hobbies. For example, individuals who engage in novel and exciting activities tend to experience more self-expansion [45]. Additionally, watching a television show, reading a brief story, or interacting with a cell phone can all be self-expansive activities [40,46,47]. Prior work has not considered whether companion animals provide a self-expanding relationship, and if so whether this is linked to well-being benefits. 

### 1.3. Perceived Pet Responsiveness and Insensitivity

Perceived partner responsiveness and its inverse, perceived partner insensitivity, have been studied extensively in human–human relationships, and are another factor that may impact the quality of human–companion animal relationships. Perceived partner responsiveness describes the extent to which individuals perceive their interaction partners as validating, understanding, and caring [48,49]. Validation refers to the feeling of being liked and appreciated within a relationship, which in turn increases feelings of security and acceptance [50]. Understanding leads to increased relational satisfaction [51], and a feeling of authenticity within the relationship [52]. Lastly, feeling cared for leads to a stronger ability to cope with stressful events within the relationship [53].

Perceived partner responsiveness has a positive impact on health, well-being, and mortality [54,55,56,57,58,59]. Couples who rate each other as more responsive report less stress and anxiety [55,56]. Perceived partner responsiveness is also associated with less daily negative affect reactivity [55,56] and decreased depression [60]. Furthermore, individuals who believe that their partners are responsive can receive support from their partners in challenging times, which buffers the impacts of stress [61,62,63]. Conversely, individuals who feel that their partners are less responsive or more insensitive to their needs report more stress and loneliness [61], and greater levels of anxiety and depression [55]. They also have increased mortality risk [4,57] and worse health and well-being outcomes [56,58]. 

There is a strong link between perceived partner responsiveness and insensitivity and well-being outcomes in human–human relationships, and qualitative research points to pet insensitivity and responsiveness, or “attunement” as important for mental health [64]. However, no prior quantitative work has examined perceived responsiveness or insensitivity in companion animals directly as a factor within the human–companion animal relationship and its impact on mental well-being.

### 1.4. Attachment

Attachment orientations have largely been studied in the context of human–human relationships and there is robust evidence that strong attachment predicts positive well-being. For example, secure and avoidant attachment traits correlate with more positive well-being outcomes [65], while attachment anxiety is associated with poorer well-being [65,66]. Furthermore, secure attachment has been demonstrated to be a predictor of well-being across age groups such as adolescence [67], young adulthood [65], and older adulthood [65,68,69], as well as during periods of transition [70]. In short, secure attachment is related to improved well-being.

Attachment has also been studied in human–animal relationships, and it is clear that people form attachments to their companion animals, with 91% of owners reporting feeling “very close” [11]. Beyond simple connection, companion animal owners often consider their companion animals to be sources of social support [71,72], which can promote well-being. Some studies indicate that strong attachment to a companion animal can improve emotional and social well-being, provide comfort, and decrease loneliness, psychological distress, and psychopathology, whereas the reverse is found for weaker companion animal attachments [14,73,74]. However, other studies indicate that higher levels of companion animal attachment predict higher levels of depression and loneliness [75,76,77]. A systematic review of the relationship between companion animal attachment and depression revealed that many studies indicate that strong companion animal attachment is linked to higher levels of depression or neutral depression outcomes, and that few indicate a link between strong attachment and lower levels of depression [78]. The divergence between attachment findings for human–human relationships versus human–companion animal relationships might reflect the focus of the companion animals literature on attachment as a “bondedness” [78]. In essence, there is inconclusive evidence for the role of companion animal attachment in well-being. 

### 1.5. Loneliness

People often believe that adopting a companion animal will alleviate loneliness [79]. However, while some studies do find that companion animal owners experience less loneliness [80,81], others find no significant difference in levels of loneliness between companion animal owners and non-companion animal owners [82,83]. A recent systematic review indicates that while companion animal ownership was not associated with lower levels of loneliness prior to the COVID-19 pandemic, during the pandemic companion animal owners experienced less loneliness, potentially indicating a buffering effect of companion animal ownership in times of stress [84].

In addition to companion animals potentially alleviating loneliness through their companionship, there is also evidence that having a companion animal can create other opportunities for positive social benefits [85], such as increased social interaction with others [11,86], and the appearance of being more likeable [87]. Higher degrees of human social support in combination with companion animal ownership can also reduce the experience of loneliness [23] and decrease social isolation [84]. 

In this study, loneliness will be treated not only as an outcome but also as a mediator. Given that higher levels of loneliness are associated with increased depression and other negative mental health symptoms [88,89,90], and loneliness has a direct effect on depression in path analysis models [91], it seems reasonable to examine both its direct and indirect relationship with well-being outcomes. 

## 2. Materials and Methods

### 2.1. Overview of Study

This project was initially completed as three separate studies (Study A, Study B, and Study C). Study A was completed as an initial study, while Study B was a replication of Study A with two changes to instrumentation to address two limitations of Study A. Study B included an additional demographic question related to income, as well as the Animal Attitudes Scale [92]. These changes were made as prior research indicates a strong relationship between a range of socio-demographic factors, such as income, and depression [93]. Additionally, past research indicates that attitudes toward animals impacts relationships between companion animals and owners [94,95].

Variability in results emerged between Studies A and B, and therefore Study C was completed to resolve the inconsistent findings. Study C was completed with the same instrumentation as Study B. Data were initially analyzed for each study separately, but because the instrumentation for all three studies was nearly identical, in this paper the data have been integrated into a single dataset with a larger sample size. Interested readers may find the individual results of Studies A, B, and C on the Open Science Framework at https://osf.io/n36h7.

### 2.2. Participants

A total of 1455 participants were recruited. Of these, ninety-six were excluded due to failing attention checks or for providing incomplete data (final *N* = 1359). See Table 1 for demographics information.

### 2.3. Procedure

Participants were recruited through Prolific Academic in exchange for GBP 1.30 (approx. USD 1.59). Inclusion criteria were: (1) over 18 years, (2) owned either a cat or dog, and (3) were living in the United Kingdom.

Study A consisted of eight questionnaires, seven attention checks, and demographics. Demographics included age, gender, type of companion animal (cat/dog), companion animal age and breed, and adoption year. Studies B and C consisted of the same eight questionnaires included in Study A, with the addition of a ninth questionnaire, the Animal Attitudes Scale [92]. Studies B and C included the same demographic questions as Study A, with the addition of a demographics question regarding income. Demographics were asked at the beginning, while attention check questions were randomized throughout. 

### 2.4. Materials

The study included the Patient Health Questionnaire (PHQ-9), the Generalized Anxiety Disorder Assessment (GAD-7), the Positive and Negative Affect Schedule (PANAS), the Revised UCLA Loneliness Scale, the Perceived Pet Responsiveness Scale, the Perceived Pet Insensitivity Scale, the Companion Animals Self-Expansion Scale (CASES), the Lexington Attachment to Pets Scale (LAPS), and the Animal Attitudes Scale. Each measure is scored cumulatively, with each participant receiving a total score for each measure.

To measure depression, we employed the nine-item Patient Health Questionnaire (PHQ-9) [96]. Each symptom question (e.g., “Little interest or pleasure in doing things”) was preceded by “Over the last two weeks, how often have you been bothered by the following problems?” answered on a four-point scale (1 = not at all, 2 = several days, 3 = more than half the days, 4 = nearly every day). Cronbach’s Alpha for the entire sample collected in Studies A, B, and C was 0.90 (Study A α = 0.88, Study B α = 0.91, Study C α = 0.91). 

To measure anxiety, we employed the seven-item Generalized Anxiety Disorder Assessment (GAD-7) [97]. Each symptom question (e.g., “Feeling nervous, anxious, or on edge”) was measured using the same scale as the PHQ-9. Cronbach’s Alpha for the entire sample collected in Studies A, B, and C was 0.92 (Study A α = 0.91, Study B α = 0.92, Study C α = 0.92). 

To measure both positive and negative affect, we used the 20-item Positive and Negative Affect Schedule (PANAS) [98]. Participants indicated the extent to which they had experienced 10 positive emotions (e.g., “enthusiastic”) and 10 negative emotions (e.g., “guilty”) over the last week (1 = very slightly or not at all, 2 = a little, 3 = moderately, 4 = quite a bit, 5 = extremely). Cronbach’s Alpha for positive affect for the entire sample collected in Studies A, B, and C was 0.93 (Study A α = 0.92, Study B α = 0.93, Study C α = 0.92) and for negative affect was 0.91 (Study A α = 0.91, Study B α = 0.91, Study C α = 0.91). 

To assess loneliness, we used the Revised UCLA Loneliness Scale [99,100]. Participants indicated the extent to which they had felt lonely (e.g., “I feel left out”) using a four-point scale (1 = never, 2 = rarely, 3 = sometimes, 4 = often). Cronbach’s Alpha for the entire sample collected in Studies A, B, and C was 0.74 (Study A α = 0.76, Study B α = 0.70, Study C α = 0.74). 

To assess perceived pet responsiveness, we developed a 24-item Perceived Pet Responsiveness Scale. We adapted this scale from the Perceived Partner Responsiveness and Insensitivity Scale for use in this study by changing all instances of “partner” to “pet” [101,102]. Participants indicated the extent to which they perceived their pet as responsive (e.g., “My pet really listens to me”; “My pet knows me well”) using a seven-point scale (1 = strongly disagree, to 7 = strongly agree). Cronbach’s Alpha for the entire sample collected in Studies A, B, and C was 0.97 (Study A α = 0.97, Study B α = 0.97, Study C α = 0.97). For analysis, each participants’ scores were added to form a cumulative perceived pet responsiveness score, which ranged from 24 to 168. Questionnaire items are available on the Open Science Framework at https://osf.io/wsrdt.

To assess perceived pet insensitivity, we developed a 24-item Perceived Pet Insensitivity Scale. This was also adapted from the Perceived Partner Responsiveness and Insensitivity Scale for use in this study by changing all instances of “partner” to “pet” [101,102]. Participants indicated the extent to which they perceived their pet as insensitive (e.g., “My pet does not accept my feelings and concerns”; “My pet is NOT attentive to my needs”) on a 7-point scale (1 = strongly disagree, to 7 = strongly agree). Cronbach’s Alpha for the entire sample collected in Studies A, B, and C was 0.97 (Study A α = 0.97, Study B α = 0.98, Study C α = 0.97). For analysis, each participants’ scores were added to form a cumulative perceived pet insensitivity score, which ranged from 24 to 168. Questionnaire items are available on the Open Science Framework at https://osf.io/nj6mt.

To assess self-expansion, we developed a 14-item Companion Animals Self-Expansion Scale (CASES). This scale was adapted from the Self-Expansion Questionnaire for use in this study by changing relational language to be pet-centric [103]. Each item (e.g., “How much does having a pet result in your having new experiences?”; “How much does your pet provide a source of exciting experiences?”) was preceded by “Answer each question according to the way you personally feel, using the following scale”, with each item answered on a seven-point scale (1 = not very much, 7 = very much). Cronbach’s Alpha for the entire sample collected in Studies A, B, and C was 0.96 (Study A α = 0.96, Study B α = 0.96, Study C α = 0.95). For analysis, each participants’ scores were added to form a cumulative self-expansion score, which ranged from 14 to 98. Questionnaire items are available on the Open Science Framework at https://osf.io/sudzn.

To assess participants’ attachment to their companion animals, we used the 23-item Lexington Attachment to Pets Scale (LAPS) [104]. Each item (e.g., “My pet means more to me than any of my friends”) was preceded by “Please rate your agreement with the following statements” and was answered on a four-point scale (1 = strongly disagree, 2 = somewhat disagree, 3 = somewhat agree, 4 = strongly agree). Cronbach’s Alpha for the entire sample collected in Studies A, B, and C was 0.94 (Study A α = 0.94, Study B α = 0.94, Study C α = 0.94).

To assess participants’ attitudes toward animals, we used the 20-item Animal Attitudes Scale (AAS) [92]. For each item (e.g., “It is morally wrong to hunt wild animals just for sport”) participants responded on a five-point scale (1 = strongly disagree, 2 = disagree, 3 = undecided, 4 = agree, 5 = strongly agree). Cronbach’s Alpha for the sample included in Study B and Study C was 0.89 (Study B α = 0.89, Study C α = 0.90).

## 3. Results

To understand the pattern of results across the datasets and identify any reliable trends, we integrated the studies into a formal integrative data analysis. Although the datasets from the three studies were highly similar, Study A included neither the demographic question related to income, nor the Animal Attitudes Scale. These missing items were approached as a multiple-multivariate missing data problem [105], with missing random data [106]. To address this problem, we employed a multiple imputation method through use of the MICE package in R [107,108]. This method was chosen due to its robustness, and because this method enabled us to generate values to replace missing values related to the AAS and income for Study A participants [109,110].

### 3.1. Correlations

Correlations between all relationship science concepts variables and well-being outcome variables are reported in Table 2. Perceived pet responsiveness correlates positively with anxiety and positive affect, and negatively with loneliness. Perceived pet insensitivity correlates negatively with positive affect, and positively with loneliness. Attachment correlates positively with depression, anxiety, and positive affect. Lastly, self-expansion correlates positively with positive affect, and negatively with loneliness.

### 3.2. Regressions

Five multilinear regressions were conducted to explore the relationship of perceived pet responsiveness, perceived pet insensitivity, attachment, and self-expansion, with depression, anxiety, affect, and loneliness, while controlling for differences in age, gender, pet type, income, and attitudes toward animals. Due to the very small number of participants identifying as non-binary, or not identifying a gender, these participants were removed from this dataset prior to conducting this analysis. As can be seen in Table 3, responsiveness predicted none of the outcome variables; insensitivity was a significant positive predictor of depression, anxiety, negative affect, and loneliness; attachment was a significant positive predictor of depression, anxiety, and loneliness, and a significant negative predictor of positive affect; and self-expansion was a significant positive predictor of positive affect, and a significant negative predictor of loneliness. Control variables of age and income were significant predictors in all models of all well-being outcome variables, and attitudes toward animals was an additional significant predictor of the models in which anxiety and loneliness were the well-being outcome variables.

### 3.3. Path Mediation Models

Four path mediation models tested the direct and indirect effects via loneliness of the predictor variables on the outcome variables. Path mediation models provide a representation of the relationship between a predictor variable, a mediator, and an outcome variable, wherein the mediator has an effect on the relationship between the predictor and outcome variables [111]. The indirect effects of insensitivity, attachment and self-expansion are statistically significant in all path mediation models, while the indirect effects of perceived pet responsiveness are not statistically significant in any path mediation model. Tables of results can be seen in Appendix A.

## 4. Exploratory Data Analysis

Although we had no hypotheses related to gender differences or companion animal-type differences, we chose to include an exploratory data analysis related to these topics. Currently, there is no existing literature related to gender differences and perceived pet responsiveness, perceived pet insensitivity, and self-expansion within human–companion animal relationships. Additionally, the existing literature provides a limited and contradictory picture related to attachment to companion animals and gender. One review of the existing literature found that women tend to display more attachment and positive attitudes toward animals than men [112], while a study of children found that girls report more strongly attached relationships to their companion animals than boys do [113]. However, other studies indicate that there are no significant differences between the genders related to attachment in both adults and children [114,115]. Furthermore, there is some research that indicates differences in mental health outcomes related to gender and companion animal ownership, with one study indicating that single women derive more mental well-being benefits from companion animal ownership than single men [116], while another study reported that women are more likely to suffer from sadness and loneliness after losing a companion animal [117]. To explore both differences in relationship science concepts as well as mental well-being outcomes between genders, independent sample t-tests were performed to compare these variables in males and females. The results displayed in Table 4 demonstrate no significant differences between females and males for any mental well-being outcome measures; however, female participants reported significantly higher perceived pet responsiveness, attachment, and self-expansion, as well as lower perceived pet insensitivity, than male participants. Due to the very small number of participants identifying as non-binary (*N* = 14), these participants were not included in this formal analysis. However, it should be noted that through examining descriptive statistics only in terms of the relationship science concepts, non-binary participants reported their companion animals as being more responsive (M = 121.14, SD = 25.28) and less insensitive (M = 55.14, SD = 25.39) than male- or female-identifying participants. Non-binary participants also reported higher self-expansion scores (M = 66.43, SD = 14.97), and higher attachment scores (M = 83.29, SD = 6.33). In terms of well-being outcomes, non-binary participants reported higher depression (M = 21.36, SD = 8.05), anxiety (M = 16.57, SD = 5.40), negative affect (M = 22.29, SD = 8.77), and loneliness (M = 15.79, SD = 3.64), while also reporting lower positive affect (M = 23.21, SD = 7.28), than participants identifying as either male or female.

We also chose to explore companion animal type differences related to relationship science concepts and mental well-being outcomes. As with gender differences, there is no past literature related to pet type in relation to perceived pet responsiveness, perceived pet insensitivity, and self-expansion within human–companion animal relationships. There is existing literature that indicates that individuals are more attached to their pet cats and dogs than companion animals of other animal types [118]. Studies also indicate that dog owners experience more attachment to their companion animals than cat owners do [118,119], and better mental well-being results [119]. To explore differences in relationship science concepts as well as mental well-being outcomes between cat owners and dog owners, two sample *t*-tests were performed to compare these variables in cat owners and dog owners. The results displayed in Table 5 demonstrate no significant difference between cat and dog owners for any mental health outcome measures, except for loneliness (dog owners reported less loneliness than cat owners), however dog owners reported significantly higher perceived pet responsiveness, attachment, and self-expansion, and lower perceived pet responsiveness than cat owners.

## 5. Discussion

Although past research has been conducted on the impact of companion animals on well-being, this research has yielded variable results [13]. The present study sought to explain the variability in these results through studying relationship science concepts within human–companion animal relationships: self-expansion, attachment, perceived pet responsiveness, and perceived pet insensitivity. Returning to our hypotheses, we posited that self-expansion, perceived pet responsiveness, and attachment would predict lower levels of depression, anxiety, and negative affect, and predict higher levels of positive affect; and we posited that perceived pet insensitivity would predict the opposite results. We also hypothesized that loneliness would mediate all relationships between relationship science concept variables and mental well-being outcome variables. Some of the results, such as the relationship between self-expansion and positive affect, supported our hypotheses. However, other results, such as the relationship between attachment and negative affect, did not. Finally, loneliness appears to be a strong mediator on the relationship between attachment and all mental well-being outcomes (contrary to our hypothesis), and a mediator to a lesser extent on the relationships between perceived pet responsiveness and self-expansion and all mental well-being outcome variables (in alignment with our hypothesis).

### 5.1. Mixed Support for Hypotheses

Perceived pet insensitivity emerged as a strong predictor variable in this study, with results of the integrative data analysis indicating that perceived pet insensitivity predicts higher levels of depression, anxiety, negative affect, and loneliness. The existing literature suggests that most people adopt a companion animal to gain companionship and emotional support [79], and so perhaps a companion animal’s perceived insensitivity to their owner could be especially distressing. This distress may therefore result in increased negative mental health symptoms due to the unmet expectations of the relationship and unmet emotional support needs. We also know from the existing human–human relationship literature that perceived partner insensitivity is associated with increased loneliness, stress, anxiety, and depression, and therefore our results align with this literature [55,61]. It stands to reason that these negative mental well-being outcomes caused by perceived partner insensitivity may also be caused by perceived pet insensitivity. 

Attachment also emerged as a strong predictor variable, positively predicting higher levels of depression, anxiety, and loneliness; and negatively predicting positive affect. These results were contrary to our hypothesis; we predicted that attachment would predict better mental well-being. Our attachment finding aligns with some of the existing human–animal literature that indicates that higher attachment to companion animals predicts higher levels of depression and loneliness [75,76,77], although the other existing literature indicates that strong companion animal attachment bonds predict better well-being outcomes [73,74]. It may be that stronger attachment to companion animals predicts worse mental well-being outcomes due to decreased human social support: if individuals are overly attached or reliant on their cat or dog for support, this may result in loss of other important forms of social support, or rather may saturate the need for alternative sources of social support. Human social support is linked to positive mental health outcomes such as lower levels of depression [120,121], and this may explain these results. Secure human attachments may also saturate the need for an attachment to a companion animal, and so human–human attachment and perceived social support are important variables to consider in future human–animal research. Alternatively, however, because this work is cross-sectional, as is much of the other work on this topic, it should also be considered that perhaps individuals with anxiety or depression may form stronger attachments to their companion animals. We cannot make such assumptions based on our findings and so future work could examine the directionality of the relationship between attachment and mental health in companion animal owners.

Results related to self-expansion were partially in agreement with our hypotheses. Self-expansion predicted positive affect. This aligns with the literature related to self-expansion and well-being, which indicates that self-expansion is enjoyable as an experience [37], and that the increased self-efficacy often associated with self-expansion also leads to better well-being outcomes [38]. Additionally, self-expansion predicted lower levels of loneliness. Although little research has been completed related to self-expansion and loneliness, one study found that individuals who experience self-expansion through their cellular phone also experience more loneliness when separated from their phone [40]. It could be posited that the presence of another human, animal, or medium that creates an opportunity for self-expansion could therefore decrease the experience of loneliness. However, it does remain surprising that self-expansion did not predict lower levels of depression, in alignment with previous research on the subject, which found that self-expansion is linked to reduced depression [39].

Lastly, although perceived pet responsiveness displayed some correlation with mental well-being outcomes (perceived pet responsiveness correlated positively with positive affect and anxiety, and negatively with loneliness), perceived pet responsiveness was not a significant predictor in any multilinear regression model. Previous research on perceived partner responsiveness within human–human relationships indicates that higher levels of perceived partner responsiveness are associated with decreased anxiety, depression, negative affect reactivity, and stress, and better overall well-being [55,57,58,60,61]. Based on the robust previous research regarding perceived partner responsiveness and well-being outcomes, it is particularly surprising that this was not observed within this study.

### 5.2. Mediating Effect of Loneliness on Mental Well-Being

Our hypotheses related to loneliness as a mediator were supported in all path mediation models except for the models in which perceived pet responsiveness was the predictor variable. The results of the path mediation models indicated that loneliness significantly mediates the relationship between perceived pet insensitivity, attachment, and self-expansion, as well as all mental well-being outcome measures.

The results related to perceived pet insensitivity may indicate that perceiving one’s companion animal as insensitive may cause an individual to feel lonelier, especially in the context of individuals who adopt a pet to have a much-desired companion [79]. The results related to attachment may indicate that more study is needed in this area, especially regarding human social support. The literature indicates that companion animal owners with lower levels of human social support experience increased levels of loneliness and other negative mental well-being outcomes [23,122]. It would be interesting to explore further whether this would explain the relationships observed in the path mediation models. Lastly, the findings related to self-expansion align with our hypotheses. Path mediation model analysis demonstrates that self-expansion leads to lower levels of loneliness, which in turn lead to lower levels of depression, anxiety, and negative affect, and higher levels of positive affect. Although there is limited research on self-expansion and loneliness, these results do align with the literature that indicates that companion animal owners may engage in activities that could be considered self-expansive, which allow them opportunities to meet and engage with others, thereby reducing loneliness [117,123].

### 5.3. Gender and Companion Animal Type Differences

Results of exploratory analysis indicated that both gender and animal type had an impact on participants’ report of attachment, self-expansion, and perceived pet responsiveness and insensitivity; but not on mental well-being outcomes. Female participants reported significantly higher perceived pet responsiveness, attachment, and self-expansion, and lower perceived pet insensitivity, than male participants. Put simply, they seemed to have more expanding and responsive relationships with their companion animals. There is no past literature related to perceived pet responsiveness and insensitivity, nor pet-induced self-expansion related to gender, and therefore these results are novel. Results related to attachment align with some of the limited literature on the subject, which indicates that women display stronger attachment to their companion animals than men [112], and that girls report more strongly attached relationships to their companion animals than boys do [113]. Interestingly, however, there were no significant differences between male and female participants in mental well-being outcomes.

Exploratory analysis also indicated that dog owners reported significantly higher perceived pet responsiveness, attachment, and self-expansion, and lower perceived pet insensitivity, than cat owners. As with gender differences, there is no past literature related to perceived pet responsiveness and insensitivity, nor pet-induced self-expansion, related to pet type. The existing literature indicates that dog owners feel more attached to their companion animals than cat owners do, and the results from this exploratory analysis align with that result [118,119]. However, literature also indicates that dog owners derive more well-being benefits than owners of other companion animals [119]. This is not reflected in the current study except for related to loneliness, which indicated that there were no significant differences in mental well-being outcomes between cat and dog owners, except for loneliness (dog owners reported less loneliness than cat owners).

### 5.4. Significance

The findings from the present research are significant for several reasons. This is the first study, to our knowledge, that has explored the impact of self-expansion, perceived responsiveness, and perceived insensitivity in human–animal relationships, which are topics widely studied in human–human relationships within the field of social psychology. It is our hope that the present research may lay groundwork for the study of these dimensions more thoroughly within human–animal relationships, in order to gain more understanding regarding the depth and intricacies of these relationships. Additionally, this study has potential usefulness clinically as it provides further background for how companion animal acquisition may be useful within the context of mental health, including issues related to overall human–companion animal compatibility and expectations regarding the human–companion animal relationship. For example, this study provides insight specifically into how important perceived pet insensitivity may be in well-being outcomes, and provides validation for individuals who may feel that having a companion animal has actually worsened their mental health.

## 6. Conclusions

The goals of the present research were to explain the variability in results of past research on human–companion relationships and well-being; to explore four of the relationship dimensions that could explain this variability; and to explore how self-expansion, perceived responsiveness, and perceived insensitivity may impact the human–companion animal relationship. We met these goals, despite our finding that all predictor variables were not significant in predicting each of the well-being outcomes. Importantly, perceived pet insensitivity was found to be a strong positive predictor of all well-being outcomes except for positive affect; attachment was found to be a strong positive predictor of depression, anxiety, and loneliness, and a strong negative predictor of positive affect; and self-expansion was found to be a strong positive predictor of positive affect and a strong negative predictor of loneliness. Furthermore, gender and companion animal type may play a role in the experience of the relationship dimension explored in this study. These results provide a potential missing link in the variability of the results of the past literature; however, more research is needed. Additionally, this study has both clinical and social psychological implications, and has laid a foundation for future research regarding further exploration of the human–companion animal relationship and the factors that may predict better well-being outcomes.

## Figures and Tables

**Table 1:** Demographics table.

	Overall (*N* = 1359)
Gender	
Female	734 (54.0%)
Male	610 (44.9%)
Non-Binary	14 (1.0%)
Prefer not to say	1 (0.1%)
Age	
Mean (SD)	40.7 (13.9)
Median [Min, Max]	38.0 [18.00, 80.0]
Depression	
Mean (SD)	15.6 (5.8)
Median [Min, Max]	14.0 [9.0, 36.0]
Anxiety	
Mean (SD)	12.6 (5.0)
Median [Min, Max]	11.0 [7.0, 28.0]
Pet Type	
Cat	587 (43.2%)
Dog	772 (56.8%)
Pet Age	
Mean (SD)	7.0 (4.3)
Median [Min, Max]	7.0 [0.0, 24.0]

**Table 2:** Correlations among depression, anxiety, positive affect, negative affect, loneliness, responsiveness, insensitivity, attachment, self-expansion, and attitudes toward animals.

	Depression	Anxiety	Positive Affect	Negative Affect	Loneliness	Responsiveness	Insensitivity	Attachment	Self-Expansion	Attitudes
Depression		0.810 ***	−0.483 ***	0.729 ***	0.592 ***	0.021	0.018	0.077 **	0.011	0.039
Anxiety			−0.415 ***	0.768 ***	0.526 ***	0.056 *	−0.005	0.100 ***	0.042	0.074 **
Positive Affect				−0.358 ***	−0.469 ***	0.178 ***	−0.171 ***	0.113 ***	0.265 ***	0.012
Negative Affect					0.509 ***	−0.007	0.041	0.015	0.015	−0.028
Loneliness						−0.064 *	0.078 **	0.012	−0.070 **	−0.074 **
Responsiveness							−0.831 ***	0.725 ***	0.672 ***	0.190 ***
Insensitivity								−0.671 ***	−0.587 ***	−0.181 ***
Attachment									0.675 ***	0.371 ***
Self-Expansion										0.173 ***
Attitudes										

*Note.* * *p* < 0.05, ** *p* < 0.01, *** *p* < 0.001.

**Table 3:** Multilinear regressions where relationship science concepts predict well-being outcomes, controlling for age, gender, and pet type.

	Depression β [95% CI]	Anxiety β [95% CI]	Positive Affect β [95% CI]	Negative Affect β [95% CI]	Loneliness β [95% CI]
(Intercept)	12.51 *** [8.20–16.81]	8.16 *** [4.40–11.92]	24.92 *** [18.60–31.24]	18.88 *** [13.33–24.42]	14.80 *** [12.43–17.17]
Responsiveness	0.01 [−0.01–0.03]	0.02 [−0.00–0.03]	0.01 [−0.02–0.04]	0.01 [−0.02–0.03]	−0.01 [−0.02–0.00]
Insensitivity	0.03 ** [0.01–0.04]	0.03 *** [0.01–0.04]	−0.03 [−0.05–0.00]	0.03 ** [0.01–0.05]	0.01 * [0.00–0.02]
Attachment	0.07 *** [00.03–0.12]	0.05 ** [0.01–0.09]	−0.11 ** [−0.17–−0.05]	0.03 [−0.02–0.09]	0.06 *** [0.04–0.08]
Self-Expansion	−0.02 [−0.04–0.01]	−0.01 [−0.03–0.01]	0.15 *** [0.11–0.18]	0.01 [−0.02–0.04]	−0.02 ** [−0.03–−0.00]
Pet Type (Dog)	−0.36 [−1.00–0.27]	−0.07 [−0.62–0.48]	−0.84 [−1.77–0.09]	−0.48 [−1.29–0.34]	−0.33 [−0.68–0.02]
Age	−0.10 *** [−0.13–−0.08]	−0.09 *** [−0.11–−0.07]	0.07 *** [0.04–0.11]	−0.14 *** [−0.17–−0.11]	−0.04 *** [−0.05–−0.03]
Gender (Male)	0.40 [−0.21–1.02]	0.03 [−0.51–0.57]	0.10 [−0.81–1.00]	0.06 [−0.73–0.86]	0.26 [−0.08–0.60]
Income	−0.53 *** [−0.76–−0.29]	−0.44 *** [−0.64–−0.23]	0.83 *** [0.49–1.17]	−0.37 * [−0.67–−0.07]	−0.40 *** [−0.53–−0.28]
Attitudes	0.02 [−0.01–0.05]	0.03 * [0.01–0.06]	−0.10 [−0.05–0.04]	−0.01 [−0.04–0.03]	−0.03 ** [−0.04–−0.01]
R^2^/R^2^ adjusted	0.083/0.077	0.087/0.081	0.110/0.104	0.073/0.067	0.081/0.075

*Note.* * *p* < 0.05, ** *p* < 0.01, *** *p* < 0.001.

**Table 4:** Two sample *t*-tests comparing differences between males and females in both relationship science concepts and mental well-being outcomes.

	Males		Females				
	Mean	SD	Mean	SD	t(df)	*p*	Range of Possible Scores [Min, Max]
Perceived pet responsiveness	102.646	29.627	109.304	30.754	t(1313.2) = −4.031	<0.001	[24, 168]
Perceived pet insensitivity	74.325	29.508	68.790	30.997	t(1317.5) = 3.346	<0.001	[24, 168]
Attachment	73.384	11.949	77.881	11.348	t(1270.8) = −7.029	<0.001	[23, 92]
Self-Expansion	58.428	17.891	61.767	18.659	t(1314.4) = −3.339	<0.001	[14, 98]
Depression	15.584	5.957	15.480	5.527	t(1257.4) = 0.329	0.742	[9, 36]
Anxiety	12.357	5.107	12.665	4.934	t(1280.2) = −1.116	0.265	[7, 28]
Positive Affect	29.131	8.736	29.149	8.368	t(1275.7) = −0.037	0.971	[10, 50]
Negative Affect	17.730	7.507	17.668	7.200	t(1276.3) = 0.153	0.878	[10, 50]
Loneliness	13.728	3.286	13.447	3.027	t(1253.2) = 1.617	0.106	[6, 24]

**Table 5:** Two sample *t*-tests comparing differences between cat owners and dog owners in both relationship science concepts and mental well-being outcomes.

	Cat Owners		Dog Owners				
	Mean	SD	Mean	SD	t(df)	*p*	Range of Possible Scores [Min, Max]
Perceived pet responsiveness	97.020	31.670	113.608	27.300	t(1155) = −10.143	<0.001	[24, 168]
Perceived pet insensitivity	81.402	30.869	63.303	27.651	t(1184.2) = 11.195	<0.001	[24, 168]
Attachment	72.993	12.674	78.154	10.592	t(1131.2) = −7.973	<0.001	[23, 92]
Self-Expansion	54.621	19.147	64.653	16.470	t(1151.2) = −10.149	<0.001	[14, 98]
Depression	15.897	5.939	15.352	5.643	t(1227.2) = 1.714	0.087	[9, 36]
Anxiety	12.693	5.038	12.468	5.027	t(1260.1) = 0.819	0.413	[7, 28]
Positive Affect	28.654	8.583	29.389	8.504	t(1255.6) = −1.569	0.117	[10, 50]
Negative Affect	18.118	7.483	17.459	7.261	t(1241.6) = 1.629	0.104	[10, 50]
Loneliness	13.874	3.237	13.387	3.086	t(1229) = 2.801	0.005	[6, 24]

## Data Availability

The data presented in this study are available on request from the corresponding author. The data are not publicly available due to privacy reasons.

## Appendix A

**Table A1:** Integrative data analysis path mediation models where relationship science concepts are regressed on loneliness, which is regressed on depression.

Direct Effects	β [95% CI]	SE	Indirect Effects	β [95% CI]	SE
Responsiveness → loneliness	−0.003 [−0.014–0.007]	0.006	Responsiveness → loneliness → depression	−0.004 [−0.015–0.008]	0.006
Insensitivity → loneliness	0.012 * [0.002–0.022]	0.005	Insensitivity → loneliness → depression	0.012 * [0.002–0.023]	0.005
Attachment → loneliness	0.049 *** [0.028–0.071]	0.011	Attachment → loneliness → depression	0.052 *** [0.029–0.077]	0.012
Self-expansion → loneliness	−0.018 ** [−0.031–−0.005]	0.007	Self-expansion → loneliness → depression	−0.020 * [−0.034–−0.005]	0.007
Responsiveness → depression	0.014 [−0.002–0.032]	0.008			
Insensitivity → depression	0.016 [0.001–0.032]	0.008			
Attachment → depression	0.033 [0.000–0.068]	0.018			
Self-expansion → depression	0.001 [−0.020–0.022]	0.010			
Loneliness → depression	1.078 *** [0.991–1.156]	0.040			

*Note.* * *p* < 0.05, ** *p* < 0.01, *** *p* < 0.001.

**Table A2:** Integrative data analysis path mediation models where relationship science concepts are regressed on loneliness, which is regressed on anxiety.

Direct Effects	β [95% CI]	SE	Indirect Effects	β [95% CI]	SE
Responsiveness → loneliness	−0.003 [−0.014–0.008]	0.006	Responsiveness → loneliness → anxiety	−0.003 [−0.012–0.006]	0.005
Insensitivity → loneliness	0.012 * [0.002–0.022]	0.005	Insensitivity → loneliness → anxiety	0.010 * [0.001–0.018]	0.004
Attachment → loneliness	0.049 *** [0.028–0.070]	0.011	Attachment → loneliness → anxiety	0.041 *** [0.023–0.060]	0.009
Self-expansion → loneliness	−0.018 ** [−0.033–−0.004]	0.007	Self-expansion → loneliness → anxiety	−0.015 * [−0.028–−0.003]	0.006
Responsiveness → anxiety	0.020 ** [0.005–0.032]	0.007			
Insensitivity → anxiety	0.018 ** [0.005–0.032]	0.007			
Attachment → anxiety	0.030 * [0.001–0.059]	0.015			
Self-expansion → anxiety	0.004 [−0.012–0.023]	0.009			
Loneliness → anxiety	0.837 *** [0.767–0.909]	0.037			

*Note.* * *p* < 0.05, ** *p* < 0.01, *** *p* < 0.001.

**Table A3:** Integrative data analysis path mediation models where relationship science concepts are regressed on loneliness, which is regressed on positive affect.

Direct Effects	β [95% CI]	SE	Indirect Effects	β [95% CI]	SE
Responsiveness → loneliness	−0.003 [−0.014–0.007]	0.006	Responsiveness → loneliness → positive affect	0.004 [−0.009–0.017]	0.007
Insensitivity → loneliness	0.012 * [0.002–0.021]	0.005	Insensitivity → loneliness → positive affect	−0.014 * [−0.025–−0.002]	0.006
Attachment → loneliness	0.049 *** [0.027–0.071]	0.011	Attachment → loneliness → positive affect	−0.059 *** [−0.086–−0.033]	0.014
Self-expansion → loneliness	−0.018 ** [−0.032–−0.004]	0.007	Self-expansion → loneliness → positive affect	0.022 ** [0.004–0.038]	0.009
Responsiveness → positive affect	0.002 [−0.027–0.032]	0.014			
Insensitivity → positive affect	−0.009 [−0.032–0.015]	0.012			
Attachment → positive affect	−0.067 * [−0.121–−0.013]	0.027			
Self-expansion → positive affect	0.127 *** [0.095–0.161]	0.017			
Loneliness → positive affect	−1.206 *** [−1.318–−1.091]	0.059			

*Note.* * *p* < 0.05, ** *p* < 0.01, *** *p* < 0.001.

**Table A4:** Integrative data analysis path mediation models where relationship science concepts are regressed on loneliness, which is regressed on negative affect.

Direct Effects	β [95% CI]	SE	Indirect Effects	β [95% CI]	SE
Responsiveness → loneliness	−0.003 [−0.014–0.007]	0.006	Responsiveness → loneliness → negative affect	−0.004 [−0.017–0.009]	0.007
Insensitivity → loneliness	0.012 * [0.001–0.021]	0.005	Insensitivity → loneliness → negative affect	0.014 * [0.001–0.025]	0.006
Attachment → loneliness	0.049 *** [0.028–0.068]	0.010	Attachment → loneliness → negative affect	0.058 *** [0.034–0.084]	0.013
Self-expansion → loneliness	−0.018 ** [−0.031–−0.004]	0.007	Self-expansion → loneliness → negative affect	−0.022 * [−0.038–−0.005]	0.008
Responsiveness → negative affect	0.014 [−0.010–0.038]	0.012			
Insensitivity → negative affect	0.016 [−0.007–0.038]	0.012			
Attachment → negative affect	−0.027 [−0.072–0.021]	0.023			
Self-expansion → negative affect	0.032 * [0.006–0.059]	0.014			
Loneliness → negative affect	1.197 *** [1.083–1.317]	0.061			

*Note.* * *p* < 0.05, ** *p* < 0.01, *** *p* < 0.001.
